# A novel immune subtype classification of ER-positive, PR-negative and HER2-negative breast cancer based on the genomic and transcriptomic landscape

**DOI:** 10.1186/s12967-021-03076-x

**Published:** 2021-09-20

**Authors:** Peiling Xie, Rui An, Shibo Yu, Jianjun He, Huimin Zhang

**Affiliations:** 1 Department of Breast Surgery, The First Affiliated Hospital of Xi’an Jiaotong University, 277 West Yanta Road, 710061 Xi’an, People’s Republic of China; 2 Department of Hepatological Surgery, The First Affiliated Hospital of Xi’an Jiaotong University, 277 West Yanta Road, 710061 Xi’an, People’s Republic of China

**Keywords:** Breast cancer, ER+/PR−/HER2−, Immune classification, Multi-omics

## Abstract

**Background:**

The diversity and plasticity behind ER+/PR−/HER2− breast cancer have not been widely explored. It is essential to identify heterogeneous microenvironment phenotypes and investigate specific genomic events driving the formation of these phenotypes.

**Methods:**

Based on the immune-related gene expression profiles of 411 ER+/PR−/HER2− breast cancers in the METABRIC cohort, we used consensus clustering to identify heterogeneous immune subtypes and assessed their reproducibility in an independent meta-cohort including 135 patients collected from GEO database. We further analyzed the differences of cellular and molecular characteristics, and potential immune escape mechanism among immune subtypes. In addition, we constructed a transcriptional trajectory to visualize the distribution of individual patient.

**Results:**

Our analysis identified and validated five reproducible immune subtypes with distinct cellular and molecular characteristics, potential immune escape mechanisms, genomic drivers, as well as clinical outcomes. An immune-cold subtype, with the least amount of lymphocyte infiltration, had a poorer prognosis. By contrast, an immune-hot subtype, which demonstrated the highest infiltration of CD8+ T cells, DCs and NK cells, and elevated IFN-γ response, had a comparatively favorable prognosis. Other subtypes showed more diverse gene expression and immune infiltration patterns with distinct clinical outcomes. Finally, our analysis revealed a complex immune landscape consisting of both discrete cluster and continuous spectrum.

**Conclusion:**

Overall, this study revealed five heterogeneous immune subtypes among ER+/PR–/HER2− breast cancer, also provided important implications for clinical translations.

**Supplementary Information:**

The online version contains supplementary material available at 10.1186/s12967-021-03076-x.

## Background

Hormone receptor (HR) positive breast cancer, a clinically and biologically heterogenous disease [1], has been categorized into two major groups, known as luminal A and B subtypes [2]. These two molecular entities have significant differences in prognosis and response to therapies [3]. In terms of endocrine sensitivity, clinical data suggest that luminal A and B tumors benefit equally, but that patients with luminal A tumors have a better baseline prognosis than those with luminal B tumors [4, 5]. On the other hand, luminal A tumors are proved less sensitive than luminal B tumors in terms of chemotherapy benefit [6]. Progesterone receptor (PR) positivity, especially high expression of PR, is more frequently observed in tumors with favorable prognosis (i.e., luminal A) than those with poor outcomes (i.e., luminal B) [7]. It is important to note that a substantial number of luminal B tumors are PR negative, albeit merely constitute ~ 10–15% of all breast cancers. Patients with estrogen receptor (ER) positive and PR-negative (ER+/PR−) tumors experienced higher risk of mortality than tumors with ER+/PR+ status [8, 9], suggesting that ER+/PR− tumors are a more aggressive phenotype and may benefit from more escalated therapies.

Efforts have been made to understand the molecular mechanisms that promote the aggressive phenotype, accompanied by the loss of PR expression. Initially, PR was recognized as the downstream gene of ER, and the lack of PR expression in ER+ tumors was considered predictive of limited endocrine responsiveness [10]. Therefore, the molecular nature of ER+/PR− phenotype was attributed to ER inactivity or low circulating estrogen. This hypothesis, however, did not fully explain why some ER+/PR− tumors remain sensitive to endocrine therapy, even respond nearly as well to anastrozole as tumors that are ER+/PR+ [5, 11]. Later, several clinical data have suggested that elevated growth factors, such as human epidermal growth factor receptor 2 (HER2), may activate the PI3K-Akt-mTOR signaling pathway, so as to shape the ER+/PR− phenotype and lead to tamoxifen resistance [12, 13]. Nevertheless, HER2 positivity rate in ER+/PR− breast cancers ranges from 15 to 20% [14], indicating that a large amount of ER+/PR− tumors are HER2-negative and their etiology may not be explained by HER2 amplification. To date, the diversity and plasticity behind ER+/PR−/HER2− breast cancer have not been widely explored.

Immunotherapy is emerging as a pillar of modern cancer treatment. Importantly, immune checkpoint inhibitors (ICIs), such as anti-PD-L1 antibodies, have demonstrated durable response and unprecedented clinical benefit across multiple solid tumors [15]. Recently, IMpassion130, the first phase III trial of ICI in metastatic triple-negative breast cancer (TNBC, ER−/PR−/HER2−) has achieved a striking clinical success [16]. The encouraging results obtained in this trial have reignited interest in immunotherapeutic approaches for breast cancer. In spite of this, the clinical experience with ICIs in HR+ breast cancer, which are more immunologically cold than its TNBC counterpart, has been marginal [17]. Considering the prevalence of HR+/HER2− breast cancer, identifying even a small subset of immunologically hot HR+/HER2− tumors could still be of great practical value. As mentioned above, the loss of PR could translate tumors with HR+/HER2− status into a more aggressive disease and poor prognosis [8, 9]. In addition, ER+/PR−/HER2− breast cancers, are proved less endocrine responsive and more chemosensitive than other luminal tumors [10]. These data raise the possibility that ER+/PR−/HER2− breast cancer should be closer to TNBC than to luminal subtype, at both clinically and biologically. Therefore, we hypothesized that ER+/PR−/HER2− breast cancer has heterogeneous microenvironment phenotypes and specific genomic events drive the formation of these phenotypes.

In this study, we classified ER+/PR−/HER2− breast tumors into five immune subtypes based on consensus clustering of immune-related gene expression profiles, and further validated their reproducibility in an independent meta-cohort. Each of the five immune subtypes was associated with distinct gene expression pattern, cellular and molecular characteristics, potential immune escape mechanisms, genomic drivers, as well as clinical outcomes.

## Methods

### Patients and datasets

The first cohort, from the Molecular Taxonomy of Breast Cancer International Consortium (METABRIC) database, consisted of 411 cases of primary operable ER+/PR−/HER2− breast cancer with gene expression, GISTIC-processed copy number variation and mutation annotation files and corresponding clinical information (downloaded from cBioPortal, http://www.cbioportal.org/) (Additional file 1: Table S1). Gene expression profiles were generated using the Illumina_Human_WG-v3 array platform and normalized by quantile normalization with linear modeling batch correction. Copy number levels were generated on the Affymetrix SNP Array 6.0 and normalized using the supervised normalization of microarrays framework and also using DNAcopy to define low- and high-level copy number thresholds. The values were defined as follows: − 2 = homozygous deletion; − 1 = hemizygous deletion; 0 = neutral/no change; 1 = gain; 2 = high level amplification. A 173-gene exome sequencing panel was used to identify somatic gene mutations and generate measures of tumor mutation burden (package maftools). The second cohort was from the University of Texas MD Anderson Cancer Center (Houston, TX, USA), including three independent datasets (GSE25066, GSE20271, GSE20194). Normalized expression microarray and clinical data for 135 ER+/PR−/HER2− breast cancer cases were obtained from the Gene Expression Omnibus (GEO, https://www.ncbi.nlm.nih.gov/geo/) database (Additional file 1: Table S1). Microarray data were generated using Affymetrix HG-U133A and normalized by MAS5 algorithm. The combat function was applied to remove the batch effects in combining the data of 135 samples from 3 studies (package sva) (Additional file 1: Figure S1). Each gene expression was transformed by log2 and z-scoring across patients in these two cohorts.

### Identification of immune subtypes and gene modules

To uncover unbiased immune subtypes, first we curated a compendium of microenvironment genes reflecting various immunological processes. We focused on two aspects in the gene selection: first, microenvironment cell-specific genes derived from two signatures, TCIA [18] and MCP-counter [19]; second, immune-related genes such as cytokines, cytokine receptors, and genes related to the antigen processing and presentation, downloaded from the ImmPort database (https://immport.niaid.nih.gov) [20]. As a result, 1480 genes measured by all platforms were selected. After constructing the immune-related gene profiles, we then applied the k-means algorithm with the Euclidean distance metric and performed 100 bootstraps each encompassing 80% patients in the METABRIC cohort. A maximum evaluated K of 8 were selected for clustering, and the cumulative distribution function and consensus matrix were used to assess the optimal K (package ConsensusClusterPlus). To identify gene modules, we also applied consensus clustering using PAM algorithm with 1-Pearson correlation distance metric, and the remaining setting and parameters were the same. We further validated the immune subtypes in an independent meta-cohort collected from GEO. The in-group proportion (IGP) was used to quantitatively measure the consistency in subtype identification at patient level in discovery and validation cohorts (package clusterRepro). Next, the genes in each gene module were annotated in terms of GO biological processes by a web-accessible database [21], Metascape (http://metascape.org). In addition, we assessed the prognostic value of immune subtypes, and combined it with available clinical and pathologic variables in multivariate Cox proportional hazard model, using overall survival (OS) and breast cancer-specific survival (BCSS) as the endpoints.

### Assessing cellular and molecular features of immune subtypes

We analyzed the relation between the immune subtypes and 34 immune-related cellular and molecular features. The composition of microenvironment cell (24 immune cells and 2 stromal cells) in the malignant tumors were evaluated by ImmuCellAI [22] and MCP-counter [19] algorithm, respectively. We also applied the univariate Cox analysis to evaluate the prognostic value of each cell subset within each immune subtype and in the whole cohort. In addition, eight molecular signatures were included. Signatures of immune and stromal cell infiltration were derived from ESTIMATE algorithm (package ESTIMATE) [23]. Macrophage regulation score was evaluated by 112 macrophage colony-stimulating factor 1 (CSF1) response genes [24]. Lymphocyte infiltration signature score was determined by a linear model based on 18 specific lymphocyte marker genes [25]. IFN-γ [26] and TGF-β [27] response signatures were defined as co-expressed gene module and the pathway activation level of TGF-β, respectively. Inflammation score was calculated as the combination of 4 genes related to inflammation initiation [28]. Signature of cytolytic activity was computed as the mean of GZMA and PRF1 gene expression [29].

### Comparison of immunogenomic indicators and enriched oncogenic pathways among immune subtypes

The breast tumor-specific potential neoantigens predicted by NetMHCpan 4.0 were available from TSNAdb [30], by which the mutation alternation file was filtered to compute the neoantigen load (gene count) in each patient (package maftools). To assess the activity of oncogenic pathways, first we constructed a compendium containing 335 genes (representing 10 oncogenic pathways) by referring to a published article [31]. Subsequently, we applied single sample gene set enrichment analysis (ssGSEA) on these genes to calculate enrichment scores for each pathway in each sample (package GSVA).

### Defining the immune landscape

Considering the dynamic nature of the immune system, we conducted the graph learning-based dimensionality reduction analysis using reduceDimension function to illustrate the intrinsic structure and distribution of individual patients (package monocle). The discriminative dimensionality reduction with trees (DDRtree) was used as dimension reduction method, and the maximum number of components was set to 2. After dimension reduction and ordering, the immune landscape was finally inferred by plot cell trajectory function.

### Statistical analysis

ANOVA or Kruskal–Wallis test were utilized to compare continuous variables, whereas chi-square or Fisher exact test were employed for the comparison of categorical variables. Survival analysis was performed by Kaplan–Meier plots, and survival differences among the clusters were compared using log-rank test. A two-sided P-value less than 0.05 was considered significant unless otherwise stated. All statistical tests and data visualization were performed with R software (version 4.0.2).

## Results

### Immune subtypes and functional gene programmes

**Fig. 1:**
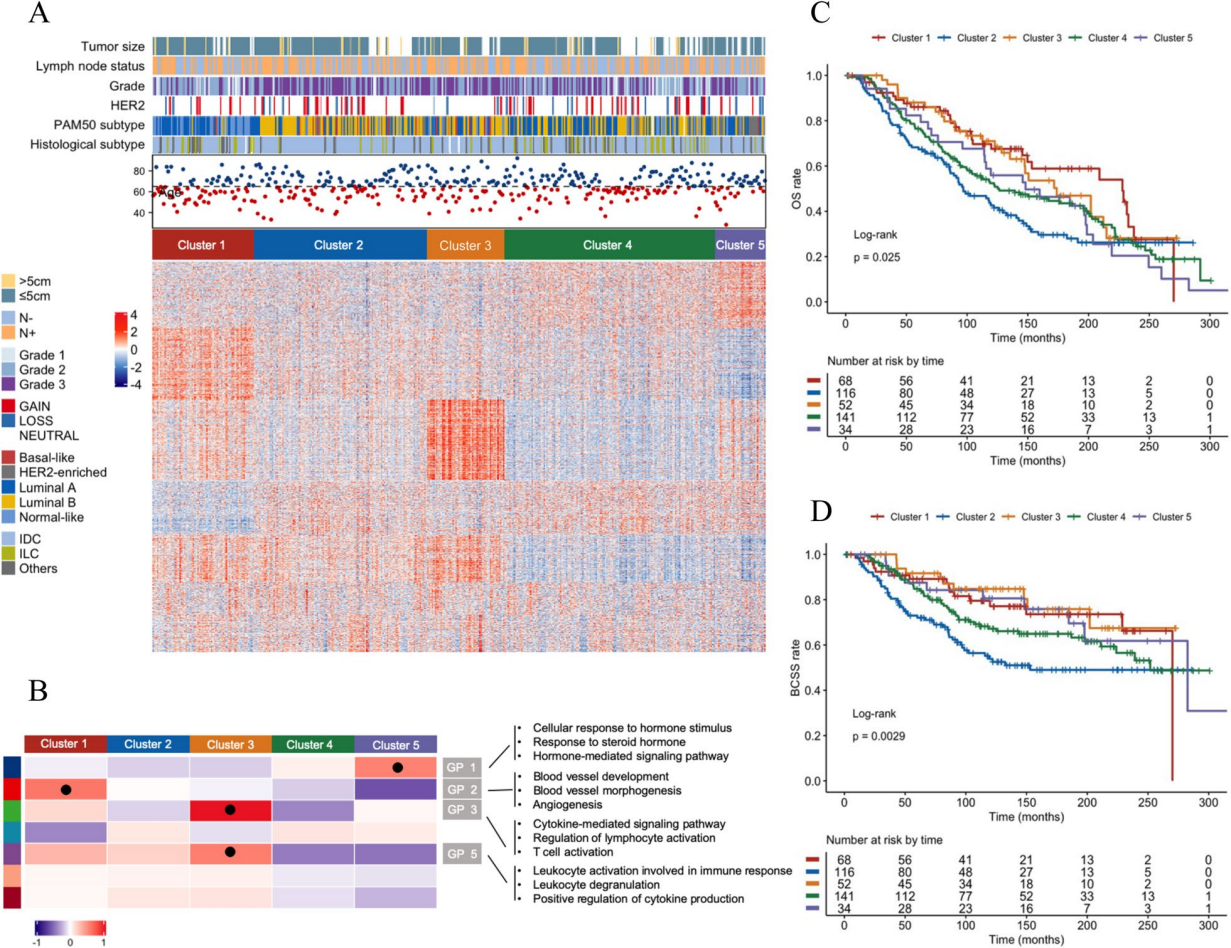
Immune subtypes and gene programmes defining ER+/PR−/HER2− breast cancer. **A** Unsupervised clustering analysis of microarray data identified five immune subtypes. **B** Heatmap of gene programmes significantly enriched in ER+/PR−/HER2− breast cancer. Scores for gene programmes (GP scores) were defined as the average expression level of all genes in a particular module. In the heatmap, colors represent mean GP scores of each cluster and black dots denote modules showing highest significance for an individual subtype. **C**, **D** Kaplan–Meier analysis of patient survival stratified by cluster: **C** OS; **D** BCSS

To facilitate visualization and uncover the underlying immune subtypes of ER+/PR−/HER2− breast cancer, first we established a comprehensive gene set that including 1480 genes representing various immunological processes (Additional file 2: Table S2). We then applied unsupervised consensus clustering on microarray data of 1480 immune-related genes for 411 ER+/PR−/HER2− tumors and identified five immune subtypes and seven gene programmes in the METABRIC cohort (Fig. 1A, B; Additional file 2: Tables S2 and S3). Among all subtypes, cluster 4 had the lowest expression in the gene programmes related to immunological processes, suggesting an immune-cold phenotype. This bore strong resemblance to cluster 5, which also lacked immunological properties but with a high expression level of cellular response to hormone stimulus programmes (GP 1). By contrast, cluster 3 had the highest expression in anti-tumor immune response programmes (GP 3), suggesting an immune-activated phenotype. Cluster 1 was characterized by elevated expression of modules involved in lymphocyte activation (GP 3), leukocyte activation (GP 5), and angiogenesis (GP 2), implying an immune-hot but suppressive microenvironment. The remaining cluster 2 demonstrated a low level of immune response (GP 3), toward an immune-inactivated phenotype (Additional file 1: Table S4). Furthermore, we utilized the expression profiles collected from GEO to validate the repeatability of clustering result (Additional file 1: Figure S3). There was moderate to good agreement between the two cohorts (IGP from cluster 1 to cluster 5: 0.90, 0.65, 0.84, 0.80 and 0.67). In brief, our analysis revealed that ER+/PR−/HER2− breast cancer had five heterogeneous phenotypes that could not be fully explained by 3-gene classifier subtype.

Given the important role of immune microenvironment in breast cancer, we investigated the clinical relevance of the immune subtypes. As depicted in Fig. 1A, the five clusters had significant differences in clinicopathological features. For instance, cluster 1 mainly consisted of luminal A subtype, while cluster 2 with higher pathological grade, was primarily made up of luminal B subtype. In addition, we observed significantly prognostic value of the immune subtypes in METABRIC cohort (OS: log-rank, P = 0.025; BCSS: log-rank, P = 0.0029) (Fig. 1C, D). Overall, cluster 2, an immune-inactivated subtype, was associated with the worst prognosis for both OS and BCSS. This survival difference was independent of age, lymph-node status and molecular subtype (Table 1). Consistent with previous studies, we found that a higher expression of immune-activated programmes was significantly associated with improved survival, even with reactive stroma.

**Table 1:** Multivariate Cox regression analysis of OS and BCSS in METABRIC cohort

Variables	OS		BCSS	
	HR (95% CI)	P-value	HR (95% CI)	P-value
Age (years)	1.04 (1.03–1.06)	4.07e−10^***^	1.00 (0.98–1.02)	0.87
Positive lymph nodes	1.07 (1.04–1.11)	4.92e−07^***^	1.09 (1.06–1.13)	3.46e−08***
PAM50 subtype				
Luminal	Ref.	–	Ref.	–
Non-luminal	0.98 (0.71–1.34)	0.89	1.09 (0.71–1.67)	0.70
Immune subtypes				
Cluster 1	0.60 (0.39–0.94)	0.024*	0.49 (0.27–0.87)	0.014*
Cluster 2	Ref.	–	Ref.	–
Cluster 3	0.59 (0.37–0.92)	0.021*	0.31 (0.16–0.62)	0.00090***
Cluster 4	0.76 (0.56–1.03)	0.075	0.65 (0.44–0.97)	0.034*
Cluster 5	0.92 (0.58–1.47)	0.73	0.56 (0.28–1.14)	0.11

Cluster 2 was used as the baseline for survival risk comparison for immune subtype variable. Luminal type was used as the baseline for survival risk comparison for PAM50 subtype.

*P < 0.05; **P < 0.01; ***P < 0.001

### Cellular and molecular characteristics of the immune subtypes

**Fig. 2:**
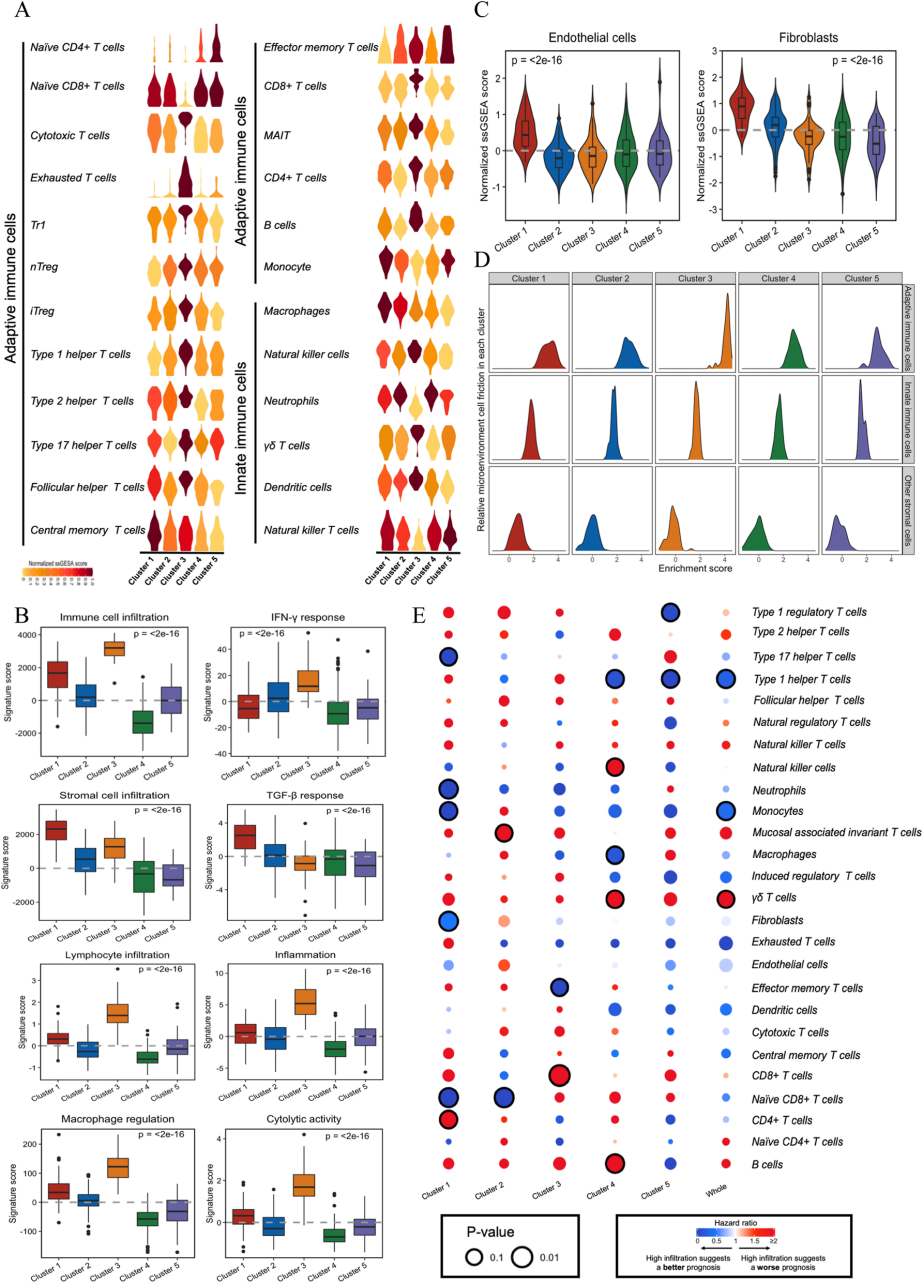
Cellular and molecular characteristics associated with immune subtypes. Enrichment scores of (**A**) 24 immune cells and (**B**) 2 stromal cells calculated by ssGSEA algorithm. In the violin plots, colors represent mean scores of each cluster (A), and the boxplot is drawn inside of violin plot (**B**). **C** Boxplots of 8 molecular signatures in each cluster. The middle bar in each box represents the median level of corresponding features in certain cluster. **D** Enrichment score distributions of three cell subsets among five clusters. **E** Prognostic value of each cell subpopulation evaluated by univariate Cox analysis for OS in whole cohort and within each cluster

We estimated the abundance of 26 subpopulations of microenvironment cells (24 immune cells and 2 stromal cells) and analyzed eight molecular signatures in each sample to systematically characterize the immune subtypes (Fig. 2A–D and Additional file 2: Table S5). Tumors in cluster 4 were characterized with relatively low degree of microenvironment cell infiltration, which was consistent with their immune-cold phenotype. Cluster 3 with immune-hot tumors was not only enriched with innate and adaptive immune cells, such as CD8+ T cells, DCs and NK cells, but also immunosuppressive cells, such as Tregs. In addition, cluster 3 had the highest IFN-γ response, inflammation and cytolytic activity signature scores. A closely related subtype is cluster 1, which also had a relatively high immune cell infiltration but with an increased stromal fraction including endothelial cells and fibroblasts, as well as the highest TGF-β response signature score. Of note, cluster 3 demonstrated the highest macrophage regulation signature score with an intermediate level of macrophage infiltration (M1-low/M2-high), suggesting a complex immune microenvironment accompanied by a shift from acute to chronic inflammation (Fig. 2A–C; Additional file 1: Figure S4). Moreover, we analyzed the relative proportion of cell subsets among all clusters by plotting the abundance distribution curve (Fig. 2D). Adaptive immune cells represented the major proportions in the microenvironments of the five clusters. The relative weight of adaptive immune cell was increased in cluster 3, whereas the relative proportion of stromal cells was increased in cluster 1. We further investigated the prognostic value of each cell subset (Fig. 2E). As a whole, a higher infiltration was associated with a more favorable outcome for most of cell subsets, even immune suppressive cells. But within each cluster, the prognostic impact of the cell subsets varied, or even in the opposite direction.

### Potential tumor immune escape mechanism of immune subtypes

The results of this study showed highly heterogenous immune subtypes in ER+/PR−/HER2− breast cancer at the cellular and molecular levels. So we got to wondering whether different subtypes had distinct tumor immune escape mechanisms. This question can be condensed into the notion of immunogenic differences, which can be classified into two aspects: extrinsic factors and intrinsic factors.

**Fig. 3:**
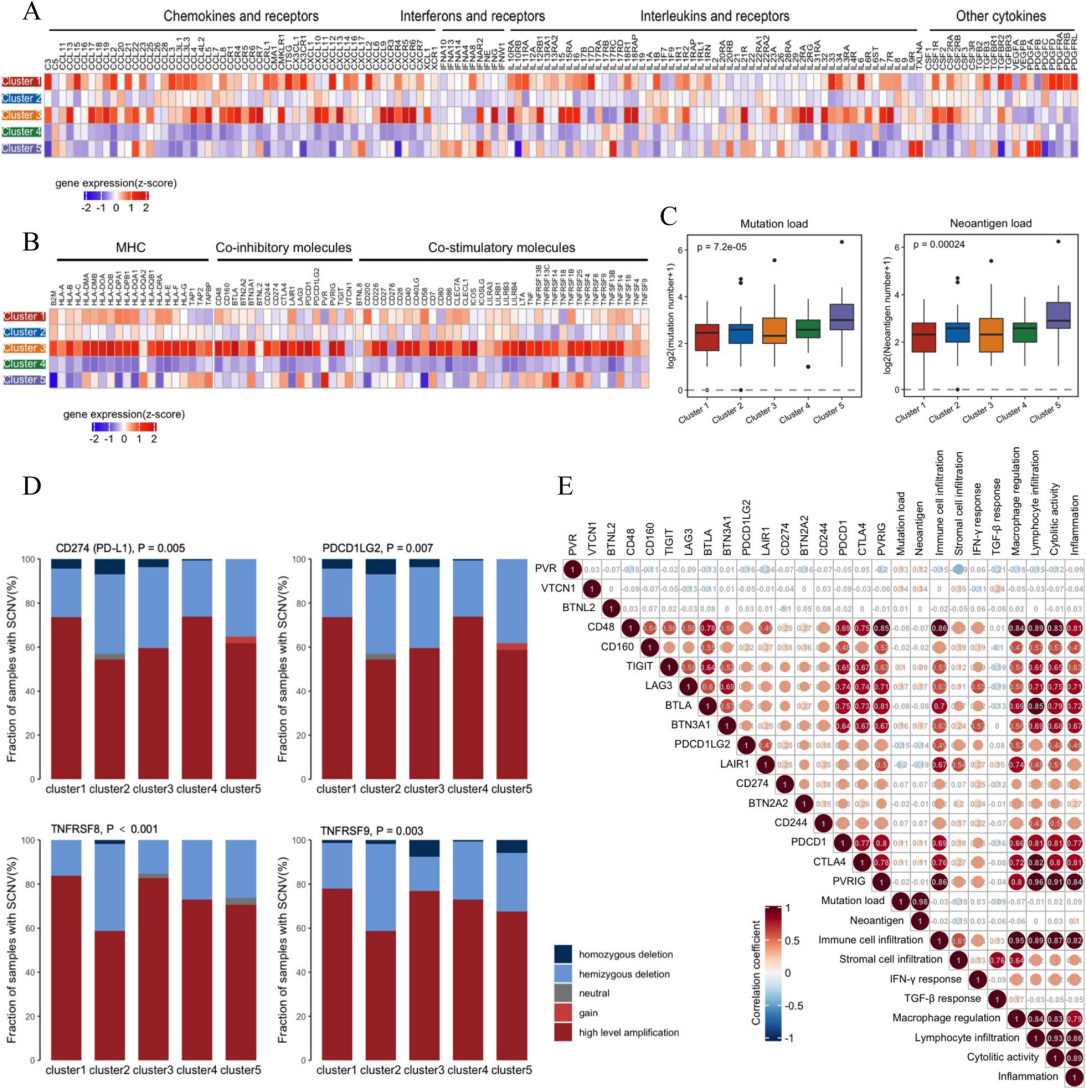
Potential tumor immune escape mechanism of immune subtypes. **A** Expression of chemokines, IFNs, ILs and other cytokines and their receptors in each cluster. **B** Expression of MHC and immunomodulatory molecules for each cluster. Expression values are represented by z-score calculated across all ER+/PR−/HER2− breast tumors. **C** Mutation load and neoantigen load in each cluster. **D** Comparison of SCNV categories of CD274, PDCD1LG2, TNFRSF8 and TNFRSF9. **E** Correlation among immune infiltration, immunogenicity and expression of immune checkpoint molecules

The extrinsic factors include infiltration of effector T cells and immunosuppressive cells, chemokines that regulate T cell recruitment and other immunomodulatory cytokines. As identified above, each cluster has its own unique pattern in the aspect of microenvironment cell infiltration. We then asked whether the expression of cytokines was consistent with the results of microenvironment cell infiltration among clusters. As depicted in Fig. 3A, cluster 3 had the highest expression of CCL5 and CXCL9, which dedicated immunoreactive and immunoresponsive tumors with increased cytotoxic T cell infiltration [32]. Also, cluster 3 had a higher concentration of immunoinhibitory cytokines, such as IL10; Cluster 1 was highly correlated with platelet-derived growth factor (PDGF) and TGF-β families, which had been proved to trigger stromal recruitment and produce additional stromal modifies, respectively, to create an immune-suppressive microenvironment [33, 34]; Cluster 2 and 5 had an intermediate expression of cytokines; and these cytokines were all relatively low in cluster 4. To sum up, the increase of immunoinhibitory factors after immune activation, the chemotaxis but inactivation of immunity, the insufficiency and inability to attract immune cells might contribute to the extrinsic immune escape of the five clusters, respectively.

The intrinsic factors included the mutation load, neoantigen load and the expression levels of MHC, co-inhibitory and co-stimulatory molecules. As shown in Fig. 3B, C, cluster 3 had the lowest tumor mutation burden and neoantigen load, but with the highest expression of MHC molecules, indicating a slight difference in immunogenicity among clusters. Additionally, we examined the expression of two class of molecules: co-stimulatory and co-inhibitory (immune checkpoint) molecules, both of which were significantly increased in cluster 3 tumors (Fig. 3B). These observations raised the question of whether the underlying genomic variants could explain the differences in the expression of co-stimulatory and co-inhibitory molecules among the clusters. We found that the elevated expression of TNFRSF8 and TNFRSF9 in cluster 1 and 3 were significantly associated with somatic copy number variations (SCNVs) (Fig. 3D). Overall, the checkpoint molecules may be upregulated to enable tumor escape after immune activation. Furthermore, we investigated the relationship among molecular signature scores, immunogenicity and the expression levels of checkpoint molecules. We found that immune infiltration signatures and the expression of most checkpoint molecules were positively correlated, whereas tumor mutation burden, neoantigen load and TGF-β response scores showed minimum correlation with these factors (Fig. 3E).

### Genomic alternation in ER+/PR−/HER2− breast cancer

**Fig. 4:**
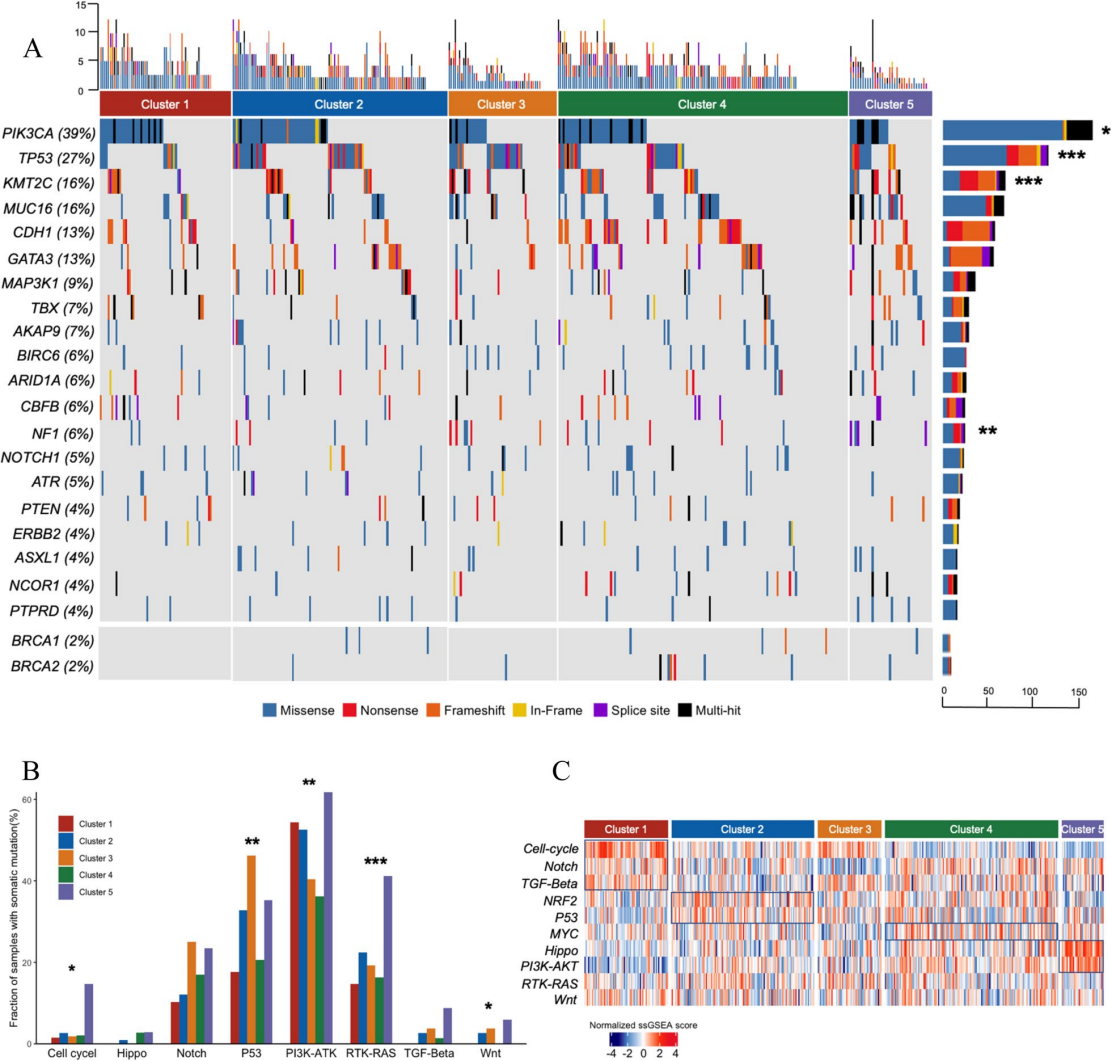
The genomic landscape of ER+/PR−/HER2− breast cancer. **A** Mutation profile is shown in column for each sample, including known cancer-related genes with top 20 mutation frequencies (top) and two recurrently homologous recombination gene in breast cancer (bottom). **B** The mutation frequencies of 10 common oncogenic pathways among clusters. **C** Heatmap of enrichment scores of 10 common oncogenic pathways among clusters. Asterisks indicate association with immune subtype (somatic mutation frequencies were tested using Fisher’s exact test. *P < 0.05, **P < 0.01, ***P < 0.001)

We made a comparison of the mutational profiles in order to pave the way to a comprehensive understanding of genomic characterization in ER+PR−HER2− breast cancer. As demonstrated in Fig. 4A, the most frequent cancer-related mutations [35] were found in PIK3CA (39%), TP53 (27%), KMT2C (16%), MUC16 (16%), CDH1 (13%), GATA3 (13%). Notably, PI3KCA, TP53 and KMT2C were differentially altered among five clusters. On the other hand, we evaluated the presence of pathologic mutation affecting homologous recombination genes, BRCA1 (2%) and BRCA2 (2%), of which there was no mutation event occurred in cluster 1. These mutated genes were further aggregated into 10 molecular mechanisms: cell-cycle pathway (CDKN1B, RB1, CCND3 and CDKN2A) in 3%; PI3K-AKT signaling (PI3KCA, AKT1, PTEN, AKT2, STK11 and PIK3R1) in 46%; Notch signaling (NCOR1, NOTCH1, NCOR2, EP300 and FBXW7) in 16%; TGF-β signaling (SMAD4 and SMAD2) in 2%; Hippo and Wnt signaling defects through NF2 and APC mutation, respectively (1%; 2%); RTK/RAS signaling (NF1, KRAS, ERBB4, ERBB2, ALK, FLK3, ERBB3, BARF and HRAS) in 24% and P53 pathway (TP53 and CHEK2) in 28%; The remaining two pathways, MYC and NRF2, had no significant mutation. We found that mutations among the Wnt, RTK/RAS, PI3K-AKT and cell-cycle pathway were most frequently observed in cluster 5, while P53 pathway in cluster 3 (Fig. 4B). By referring to the published signatures [31], we further calculated the enrichment scores of 10 common oncogenic pathways among the five clusters (Fig. 4C). The cell-cycle, Notch and TGF-β pathways were enriched in cluster 1 (all P < 0.001); The NRF2 and P53 pathways were upregulated in cluster 2 (all P < 0.01); The score of MYC pathway was increased in cluster 4 (P < 0.001); The Hippo and PI3K-AKT pathways were significantly higher in cluster 5 (all P < 0.001). Gene Set Enrichment Analysis validated some of these results (Additional file 1: Figure S5). Of note, cluster 3 with immune-activated tumors lacked specific oncogenic pathway in this study, suggesting that specific genomic alternations had the potential to induce immune-cold or immune-inactivated phenotype.

### Immune landscape of ER+/PR−/HER2− breast cancer

**Fig. 5:**
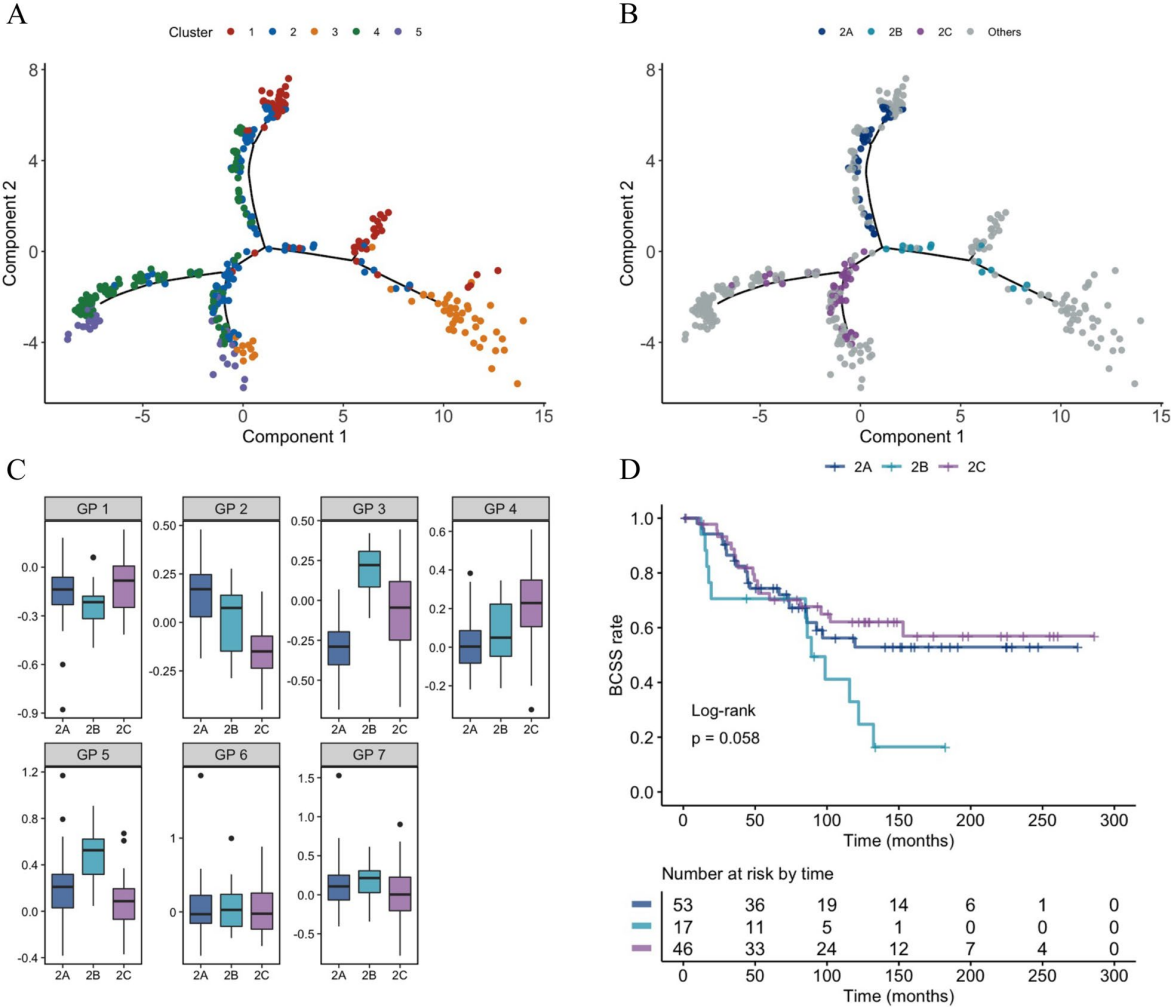
The immune landscape of ER+/PR−/HER2− breast cancer. **A** The immune landscape: each point represents a patient with colors corresponding to the immune subtype identified previously. **B** Patients of cluster 2 could be further stratified into three subgroups based on their location in the immune landscape. **C** Gene programme expression patterns were shown to illustrate the intra-cluster heterogeneity of cluster 2. **D** The three subgroups of patients stratified by the immune landscape in cluster 2 were associated with distinct prognosis

Using the immune gene expression profiles, we constructed a transcriptional trajectory to reveal the underlying structures of the distribution of individual patients, and find key gene expression programmes governing the tumor progression. Indeed, transcriptive states in the trajectory revealed the dynamics of immune process (Fig. 5A). Firstly, patients were located in separate trajectory branches, making their distinct characteristics of tumor immune microenvironment in the corresponding subtype. For instance, the immune-hot cluster 3 and immune-cold cluster 4 were observed to be distinctly positioned at the opposite end of horizontal axis in the immune landscape. Therefore, we assumed that the horizontal axis in the immune landscape may reflect the overall immune infiltration. Secondly, patients with certain immune subtype, formed two or more branched structures, revealing significant intra-cluster heterogeneity within each subtype. For instance, cluster 2 could be further segregated into three subgroups based on their location in the immune landscape, which showed different immune gene expression profiles in specific modules (Fig. 5B, C). Similar results were also observed in other immune subtypes (Additional file 1: Figure S6). Interestingly, the three subgroups of patients stratified by immune landscape in cluster 2 demonstrated distinct prognosis, although not statistically significant (BCSS: log-rank, P = 0.058) (Fig. 5D). The subset of patients with favorable outcomes (2C) was associated the lowest angiogenesis module (GP 2). Overall, these results indicated that our immune landscape analysis provided complementary value to previously identified immune subtypes.

## Discussion

Here, we used an analytical strategy to present comprehensive characterization of five reproducible immune subtypes within ER+/PR−/HER2− breast cancers in a multicohort retrospective study (Additional file 1: Figure S7). We found that each of the immune subtypes was associated with distinct gene expression modules, and demonstrated widely different patterns in tumor microenvironment cell infiltration, molecular signatures, tumor immunogenicity, regulation of immunomodulators, genomic alterations, and, importantly, prognostic efficacy. Moreover, using the graph learning-based dimensionality reduction analysis, we defined immune landscape to facilitate visualization of the distribution of individual patients. Collectively, this study recapitulates key features of ER+/PR−/HER2− breast cancer from an immunogenomics perspective, and may has important implications for clinical translation.

In recent years, accumulating data support a key role of the tumor immune microenvironment on long-term survival in patients with breast cancer [36]. Our study revealed that ER+/PR−/HER2− breast cancers of cluster 3 had the highest level of infiltration by immune effectors such as CD8+ T and NK cells, and elevated expression of IFN-γ response and cytolytic activity signatures. Accordingly, patients in cluster 3 had a favorable prognosis. By contrast, tumors of cluster 4 were characterized with reduced lymphocyte infiltration and thus these patients had relatively worse prognosis. These results are in accordance with previous studies showing higher levels of tumor infiltrating lymphocytes predict improved outcomes across cancer types. Moreover, the relative dominance between immune stimulatory and suppressive factors is also critical in determining prognosis. In our study, tumors of cluster 1 demonstrated a high level of immune cell infiltration, closely following cluster 3, but its immune composition was dominated by macrophages with increased immune-suppressive factors such as TGF-β signaling and reactive stroma. Interestingly, these two subtypes above showed no significant difference in prognosis, which may be attributed to the elevated immunoinhibitory factors after immune activation in cluster 3, thus weakening the dominant position of immune stimulation. Overall, these data suggested that the immune profiles played crucial roles in determining outcomes and could potentially stretch into future biomarker-based risk stratification strategy. Currently, the individual-based models, which requires the response to therapy and clinical outcomes to be known for each individual patient, is a subject of intense research. In comparison, our approach is gene expression-based, which reveals the underlying structures of the immune landscape within tumors and identifies the intrinsic immune subtypes with distinct outcomes. Given that this strategy is not yet able to provide clinician with improved tools for decision making, it is reasonable to imagine building a hierarchical model [37], first stratifying patients into subgroups to reduce the major inter-tumor molecular diversity and then applying subtype-specific biomarker panel to accurately predict prognosis within each subtype. Recent studies revealed that molecular-subtype-specific biomarkers have improved prediction of prognosis in many malignancies. Therefore, extended subtyping framework, combining subtyping and individual-based model could be of great practical value in biomarker-based risk stratification.

The strategy of using monoclonal antibodies against co-inhibitory receptors, termed ICI, is being used to treat an increasing number of malignancies in clinical practice or ongoing trials [16]. Unfortunately, the clinical efficacy of ICI as monotherapy has been limited to a subset of patients, PD-L1 inhibition with response rate of 20% or less in many cancer types such as breast cancer [38]. Several markers including predictive biomarkers such as PD-L1 expression by tumor cells [16, 39], tumor mutation burden [40] and DNA mismatch-repair deficiency [41] have thus been proposed to expand enrollment of patient populations that are responsive to immunotherapy. Not only that, there are ongoing efforts to develop therapeutic strategies to enhance and broaden the anti-tumor activity by using combination therapy [38]. Our approach to deeply mine cancer transcriptome and genomic data revealed a number of associations suggesting important biological conclusions with potential implications for cancer immunotherapy with ICI as monotherapy, as combination therapy with targeted agents, and for therapeutic vaccination. For patients with immune-hot tumors and elevated expression of immune checkpoint molecules (i.e., cluster 3), ICI might be used to enhance the preexisting antitumor immunity as early as possible. However, for patients in other subtypes, ICI alone might be insufficient due to the inability to induce immune activation or the presence of immune-suppressive mechanisms. For those with immune-cold tumors (i.e., cluster 4), ICI should be optimally combined with other treatment strategies such as chemotherapy and radiation therapy, which indicated a potential role in eliciting antitumor immune response. Because cluster 2 had a low immune response signal but with an intermediate level of lymphocyte infiltration and IFN-γ response, combination of ICI with co-stimulatory molecules such as CD137 and CD134 might be complementary strategies to enhance immune response [42]. The restoration of abnormal oncogenic pathways for patients in cluster 5, such as AKT-PI3K-mTOR, might be promising strategies for combining immunotherapeutic approaches. For the remaining patients in cluster 1, depending on their immune-suppressive microenvironment with active stroma, anti-TGFβ or anti-angiogenesis therapy might be used together with ICI to revert the ineffective antitumor immune response. Most importantly, we defined the immune landscape, which revealed previously unappreciated intra-cluster heterogeneity with potential clinical relevance. For instance, a fraction of patients in cluster 2 were shown to have an inferior prognosis relative to others in the same immune subtypes. This raises the question of how to optimally modulate the immune response so that patients are mobilized towards more favorable states.

Efforts to elucidate cancer genetic program in shaping immune responsiveness and its relevance to diseases are increasing [43]. Recent studies in lung cancer demonstrated that TP53 mutation represented a state of adaptive immune resistance and a high immunogenicity, which contributed to a probable sensitivity to PD-1 blockade [44, 45]. In breast cancer, mutations of TP53 were enriched in the immune favorable phenotype, in the analysis of The Cancer Genome Atlas (TCGA) datasets [46]. When the analysis was performed in each intrinsic molecular subtype, TP53 mutations were associated with the expression of immune related genes in luminal tumors [47]. These notions, to some extent, support our finding that TP53-mutated tumors were remarkably enriched in cluster 3, accompanying with increased PD-L1 expression, facilitated CD8+ T cell infiltration and augmented immunogenicity. In addition, the relationship between enrichment of oncogenic pathways and the prognostic and predictive role of immune phenotypes, was observed in an TCGA pan-cancer study [48]. Similarly, our study revealed an immune-cold phenotype of cluster 4, characterized by the lowest lymphocyte infiltration and the highest MYC pathway enrichment. Evidence from clinical and experimental studies revealed that some intrinsic pathways, such as activation of MYC, impaired T cell recruitment through failed accumulation or activation of antigen-presenting cells [49, 50]. Therefore, we speculated that the low innate immune cell chemotaxis induced by MYC might be the reason for the poor immune infiltration in cluster 4. Strategies to inhibit MYC signal might potentially convert a non-T cell inflamed tumor into a T cell inflamed tumor, importantly, improve outcomes. Overall, beyond clinical implications, the results from this study also have important biological insights into the relationship between oncogenic states and intra-tumoral immune response in ER+/PR−/HER2− breast cancer.

Limitations of our study include its retrospective nature, although we have incorporated another independent dataset for validation of our immune subtypes. In addition, the immune subtype assay is based on the gene expression profiles, which are not readily available in clinical practice as a result of cost, turnaround times and requirement of bioinformatics expertise. The surrogate definition of each immune subtype developed using specific markers could be of great practical values. Thus, we have recently designed a prospective clinical study, aiming to further validate the immune subtypes, develop the biomarker-based surrogate definitions and finally achieve the purpose of clinical translation.

## Conclusions

In summary, we identified five reproducible immune subtypes of ER+/PR−/HER2− breast cancer with distinct molecular characteristics and genomic alternations. Our research provides theoretical supports for combined therapeutic strategies in the future and offers optimal selection of patients treated with immunotherapy, so as to improve the outcomes of ER+/PR−/HER2− breast cancer.

## Supplementary Information

**Additional file 1: Fig. S1.** Batch effect evaluation after “combat” of GEO datasets. **Fig. S2.** Consensus matrix and cumulative distribution function derived from consensus clustering analysis in METABRIC cohort. **Fig. S3**. The validation of immune subtypes in the GEO cohort. **Fig. S4**. Signature scores of M1 and M2 macrophages infiltration among immune subtypes. **Fig. S5.** Gene set enrichment analysis of enriched pathways in each immune subtype. **Fig. S6.** The intra-cluster heterogeneity revealed by the immune landscape. **Fig. S7.** Workflow of this study. **Table S1.** Clinicopathological features for patients in the discovery and validation cohorts. **Table S4.** Functional enrichment analysis of gene programmes.

**Additional file 2: Table S2.** A comprehensive gene set that including 1480 genes. **Table S3.** The immune subtype annotation of each patients in the METABRIC cohort. **Table S5.** List of 8 immune molecular signatures.

## Data Availability

The data used and analyzed during the current study are available from the Molecular Taxonomy of Breast Cancer International Consortium (METABRIC) (downloaded from cBioPortal, http://www.cbioportal.org/) and Gene Expression Omnibus (GEO) (https://www.ncbi.nlm.nih.gov/geo/).

## Abbreviations

HR: Hormone receptor
PR: Progesterone receptor
ER: Estrogen receptor
HER2: Human epidermal growth factor receptor 2
ICI: Immune checkpoint inhibitor
TNBC: Triple-negative breast cancer
METABRIC: Molecular Taxonomy of Breast Cancer International Consortium
GEO: Gene Expression Omnibus
OS: Overall survival
BCSS: Breast cancer-specific survival
CSF1: Colony-stimulating factor 1
ssGSEA: Single sample gene set enrichment analysis
SCNVs: Somatic copy number variations
TCGA: The Cancer Genome Atlas
