# Supplementary material for: A novel immune subtype classification of ER-positive, PR-negative and HER2-negative breast cancer based on the genomic and transcriptomic landscape
Source: J Transl Med. 2021 Sep 20;19:398. doi: 10.1186/s12967-021-03076-x (PMC8454077; doi:10.1186/s12967-021-03076-x)
Supplement: Supplementary file 1 — Additional file 1: Fig. S1. Batch effect evaluation after “combat” of GEO datasets. Fig. S2. Consensus matrix and cumulative distribution function derived from consensus clustering analysis in METABRIC cohort. Fig. S3. The validation of immune subtypes in the GEO cohort. Fig. S4. Signature scores of M1 and M2 macrophages infiltration among immune subtypes. Fig. S5. Gene set enrichment analysis of enriched pathways in each immune subtype. Fig. S6. The intra-cluster heterogeneity revealed by the immune landscape. Fig. S7. Workflow of this study. Table S1. Clinicopathological features for patients in the discovery and validation cohorts. Table S4. Functional enrichment analysis of gene programmes. [file 12967_2021_3076_MOESM1_ESM.docx]

**Additional file 1**

**Supplementary tables**

**Table S1**. Clinicopathological features for patients in the discovery and validation cohorts

|  | **METABRIC cohort** | **GEO meta-cohort** |
| --- | --- | --- |
| Total number | 411 | 135 |
| Median age (IQR) | 65.4 (58.3-72.3) | 53.0 (44.0-60.5) |
| Tumor size (%) |  |  |
| ≤5cm | 280 (68.1) | 80 (59.3) |
| >5cm | 33 (8.0) | 55 (40.7) |
| NA | 98 (23.8) | 0 (0) |
| Lymph node status (%) |  |  |
| N- | 202 (49.1) | 36 (26.7) |
| N+ | 209 (50.9) | 99 (73.3) |
| Histological type (%) |  |  |
| IDC | 307 (74.7) | - |
| ILC | 47 (11.4) | - |
| Others | 53 (12.9) | - |
| NA | 4 (1) | - |
| Grade (%) |  |  |
| 1 | 32 (7.8) | 9 (6.7) |
| 2 | 176 (42.8) | 68 (50.4) |
| 3 | 190 (46.2) | 52 (38.5) |
| NA | 13 (3.2) | 6 (4.4) |
| PAM50 subtype (%) |  |  |
| Luminal A | 151 (36.7) | 21 (15.6) |
| Luminal B | 153 (37.2) | 21 (15.6) |
| HER2-enriched | 34 (8.3) | 7 (5.2) |
| Basal-like | 18 (4.4) | 13 (9.6) |
| Normal-like | 53 (12.9) | 10 (7.4) |
| NA | 2 (0.5) | 63 (46.7) |

‘NA’ and ‘-’ represent the corresponding information is not available.

**Table S4**. Functional enrichment analysis of gene programmes.

| **GP** | **# of genes** | **Top 5 enriched biological processes** |
| --- | --- | --- |
|  |  | GO:0032870~cellular response to hormone stimulus |
|  |  | GO:0045596~negative regulation of cell differentiation |
| 1 | 252 | GO:0040008~regulation of growth |
|  |  | GO:1903530~regulation of secretion by cell |
|  |  | GO:0048545~response to steroid hormone |
|  |  |  |
|  |  | GO:2000147~positive regulation of cell motility |
|  |  | GO:0040017~positive regulation of locomotion |
| 2 | 272 | GO:0051272~positive regulation of cellular component movement |
|  |  | GO:0001568~blood vessel development |
|  |  | GO:0030335~positive regulation of cell migration |
|  |  |  |
|  |  | GO:0019221~cytokine-mediated signaling pathway |
|  |  | GO:0046649~lymphocyte activation |
| 3 | 303 | GO:0002250~adaptive immune response |
|  |  | GO:0042110~T cell activation |
|  |  | GO:0050865~regulation of cell activation |
|  |  |  |
|  |  | GO:0019221~cytokine-mediated signaling pathway |
|  |  | GO:0045786~negative regulation of cell cycle |
| 4 | 208 | GO:0048863~stem cell differentiation |
|  |  | GO:0071456~cellular response to hypoxia |
|  |  | GO:0036294~cellular response to decreased oxygen levels |
|  |  |  |
|  |  | GO:0002274~myeloid leukocyte activation |
|  |  | GO:0002366~leukocyte activation involved in immune response |
| 5 | 181 | GO:0002263~cell activation involved in immune response |
|  |  | GO:0043299~leukocyte degranulation |
|  |  | GO:0002275~myeloid cell activation involved in immune response |
|  |  |  |
|  |  | GO:0019221~cytokine-mediated signaling pathway |
|  |  | GO:0050731~positive regulation of peptidyl-tyrosine phosphorylation |
| 6 | 123 | GO:0006935~chemotaxis |
|  |  | GO:0042330~taxis |
|  |  | GO:0002521~leukocyte differentiation |
|  |  |  |
|  |  | GO:0019221~cytokine-mediated signaling pathway |
|  |  | GO:0033674~positive regulation of kinase activity |
| 7 | 141 | GO:0007169~transmembrane receptor protein tyrosine kinase signaling pathway |
|  |  | GO:0032956~regulation of actin cytoskeleton organization |
|  |  | GO:0051347~positive regulation of transferase activity |

**Supplementary Figures**

**Fig. S1.** Batch effect evaluation after “combat” of GEO datasets.

**Fig. S2.** Consensus matrix and cumulative distribution function derived from consensus clustering analysis in METABRIC cohort.

(A) ER+/PR-/HER2- breast cancer patients. (B) immune-related genes.

**Fig. S3**. The validation of immune subtypes in the GEO cohort.

(A) Consensus matrix and cumulative distribution function for patients. (B) Unsupervised clustering analysis of gene expression profiles identified five immune subtypes.

**Fig. S4**. Signature scores of M1 and M2 macrophages infiltration among immune subtypes.

**Fig. S5.** Gene set enrichment analysis of enriched pathways in each immune subtype.

Significant enriched pathways (NOM.p-val < 0.05) in Cluster 1 (A), Cluster 2 (B), Cluster 3 (C), Cluster 4 (D), Cluster 5 (E).

**Fig. S6.** The intra-cluster heterogeneity revealed by the immune landscape.

Patients of immune subtype 1 (A), 3 (B), 4 (C) and 5 (D) could be further divided into 2 or 3 subgroups based on their location in the immune landscape.

**Fig. S7.** Workflow of this study.

s
